# Acknowledgement to Reviewers of *Journal of Clinical Medicine* in 2016

**DOI:** 10.3390/jcm6010008

**Published:** 2017-01-11

**Authors:** Journal of Clinical Medicine Editorial Office

**Affiliations:** MDPI AG, St. Alban-Anlage 66, 4052 Basel, Switzerland; jcm@mdpi.com

The editors of *Journal of Clinical Medicine* would like to express their sincere gratitude to the following reviewers for assessing manuscripts in 2016. 

We greatly appreciate the contribution of expert reviewers, which is crucial to the journal’s editorial process. We aim to recognize reviewer contributions through several mechanisms, of which the annual publication of reviewer names is one. Reviewers receive a voucher entitling them to a discount on their next MDPI publication and can download a certificate of recognition directly from our submission system. Additionally, reviewers can sign up to the service Publons (https://publons.com) to receive recognition. Of course, in these initiatives we are careful not to compromise reviewer confidentiality. Many reviewers see their work as a voluntary and often unseen part of their role as researchers. We are grateful to the time reviewers donate to our journals and the contribution they make. 

If you are interested in becoming a reviewer for *Journal of Clinical Medicine*, see the link at the bottom of the webpage http://www.mdpi.com/reviewers.

The following reviewed for *Journal of Clinical Medicine* in 2016:
Abejuela, Harmony RaylenHu, Wen-LongPradelli, LorenzoAlbano, EmanueleHuang, YuProdam, FlaviaAlvarez, VincentHung, Jan-jongRade, Jeffrey J.Andonegui, JoséIinuma, HisaeRamanathan, SudarshiniAppleton, KatherineIl’yasova, DoraRamsey, VincentAthyros, VasiliosImamura, TeruhikoRauch, BernhardAustin, BrianIshikawa, San-eRawat, Vijay P. S.Azzolini, ClaudioJames, VictoriaRhodes, JonathanBarbieri, ElenaJayaraman, SundararajanRichards, Philip ScottBartolini, LucaJeong, Hwan-JeongRimehaug, TormodBelchamber, KylieJeong, HyeoncheolRitchie, Elspeth CameronBello-Espinosa, Luis E.Jhanwar-Uniyal, MeenaRogers, Lynette K.Bendall, SarahJo, Helen E.Rojas, MauricioBillman, George E.Johnston, Donna L.Røsjø, HelgeBlack, Esther P.Jones, Joshua A.Rudkowska, IwonaBlandino, GiovanniJoonho, SeoRutenberg, MichaelBlomberg, JonasKanai, MasashiSadik, RiadhBonanno, ElenaKanasaki, KeizoSaitoh, MasaoBonay, MarcelKang, ChenSanchez-Solis, ManuelBrabletz, ThomasKapus, AndrásSaulsbury, MarilynBrown, Anthony M. C.Keklikoglou, IoannaSavagner, PierreBrumeanu, Teodor DoruKim, Mee KumSavji, NazirBuitendijk, GabrielleKim, Seok JinSchiff, MiriamByrnes, Catherine A.King, Christopher S.Schmidt, MortenCalorini, LidoKiser, Laurel J.Schmitt, UlrichCamps, CarlosKitazawa, SoheiSchoch, OttoCarnés, JerónimoKoelzer, Viktor HendrikScholl, DeanCarod Artal, Francisco JavierKok, WouterSeedat, SorayaCarter, Wayne G.Konishi, HirotakaSegal, Anthony W.Caspi, YaelKotanidou, AnastasiaSeinfeld, SyndiCastelli-Gair Hombria, JamesKothari, Anai N.Sejling, Anne-SophieCentanni, ManuelaKovacic, Jason C.Sellares, JacoboChabé, MagaliKovacs, KalmanSenechal, MarioChai, Yoke ChinKoyama, YuSgaragli, GiampietroChalela, RobertoKrapf, RetoShaffner, Donald H.Chang, Shun-FuKretschmer, KarstenShen, Jian-GangChaudhry, Ammar A.Krysinska, KarolinaShi, Zhi-YuanChavakis, EmmanouilKumar, SajeeshSoeters, Maarten R.Chen, QiKurdowska, Anna K.Spagnolo, PaoloChilds, BelindaLalonde, FrancoisStahlman, ShaunaChipps, JenniferLanger, DanielStendahl, OlleCho, NamhoonLappas, CourtneySterns, Richard H.Cohen, Michael V.Lau, Edmund M. T.Stewart, Lamonica V.Correale, MicheleLavie, Carl J.Sukocheva, OlgaCosta, Maria do CéuLavine, KorySuzuki, SatoshiDale, RussellLebaron, RichardSvennevig, KatjaDalenga, TobiasLee, Heung-ManSyn, Wing-KinDanielpour, DavidLee, Jae-HoTarantino, GiovanniDe Meirleir, KennyLee, Jung WeonTasker, Robert C.De Pablo, AliciaLee, Victor Ho FunTataranno, Maria LuisaDe Paiva, Cintia S.Levraut, JacquesTettenborn, BarbaraDe Winter, Robbert J.Liatis, StavrosThedieck, KathrinDeCarolis, Douglas D.Link, AlexanderTheret, NathalieDegrassi, FrancescaLiu, PengdaThoene, JessDemaerschalk, Bart M.Lou, Tsung-linThoma, MyriamDeng, PengLudwig, HeinzThomson, PaulaDesselberger, UlrichLyons, Paul E.Trebicka, Jonel K.Dhasarathy, ArchanaLyons, Todd W.Tripodi, MarcoDi Masi, AlessandraMahgoub, AmarTsai, JamesDonnarumma, GiovannaMarshall, JohnTsuji, YoshihisaDuffy, Shantel L.Martin, FinianTsuruda, ToshihiroDugast, PascaleMathur, HarshTurnbull, DavidDunphy, KarenMckinstry, BrianUeki, SatoshiEide, Ivar A.Medoff, Deborah R.Ulivi, PaolaEiroa-Orosa, Francisco JoseMelanitou, EvieUngefroren, HendrikElbe, DeanMelisi, DavideUysal, UtkuElison, JeffMeyerholz, DavidVan Der Jagt, MathieuEngelhardt, MonikaMiller, JessicaVan Dyke, Mark E.Esteve, JordiMithoefer, Michael C.Van Etten, JamieFabregat, IsabelMora, JorgeVan Leeuwen, Paul A. M.Farber, Harrison W.Moran, OnofreVaziri, Nosratola D.Fennell, KateMoreno-Bueno, GemaVenuti, AldoFerrari, RobertoMuñoz-Chápuli, RamónVerbraecken, JohanFlotte, Terence R.Murray, HannahViña, JuanFrisch, Steven M.Nadler, Evan P.Von Schacky, ClemensGaynor, KeithNakamura, AkioVujanovic, Anka A.Gerber, MarietteNakayama, MasayukiWahlberg, LawrenceGin-Sing, WendyNashef, LinaWalenga, Jeanine M.Gloria, AntonioNaviglio, SilvioWallis, AmyGow, Rachel V.Nehler, Mark R.Walters, Eugene HaydnGreenough, AnneNemunaitis, JohnWang, YumengGullberg, DonaldNeymotin, FlorenceWard, DouglasGulliver, Suzy BirdNie, JinleiWaris, GulamGupta, Smiti V.Norata, DaniloWary, Kishore K.Hähnel, StefanNorgren, LarsWatabe, TetsuroHammersley, RichardObermüller, NicholasWatz, HenrikHan, JialiOhji, GohWilliams, Joah L.Hanai, Jun-ichiOkura, TakafumiWollin, LutzHardman, W. ElaineO’mahony, SiobhainWu, Chung-HsinHatano, MasaruPapoff, PaolaWu, Wen-ShengHayashi, YuichiroPatrignani, PaolaYoong, MichaelHeid, IrisPenha-Goncalves, CarlosYoong, MichaelHendrix, Mary J. C.Petrakis, IsmeneYoshida, ToshimichiHiguchi, TakakazuPinnock, HilaryYoung, Alvin L.Hocker, Sara E.Pistilli, EmidioZaheer, AsgarHolland, Kathryn J.Plante, ThomasZoladz, Phillip R.Holst, JensPlaza-Díaz, JulioZöller, MargotHong, YonggeunPlomgaard, Peter
Hourani, Laurel L.Poole, David C.


